# An Overview of the Prediction of Protein DNA-Binding Sites

**DOI:** 10.3390/ijms16035194

**Published:** 2015-03-06

**Authors:** Jingna Si, Rui Zhao, Rongling Wu

**Affiliations:** Center for Computational Biology, National Engineering Laboratory for Tree Breeding, College of Biological Sciences and Technology, Beijing Forestry University, Beijing 100083, China; E-Mails: ruizhao@bjfu.edu.cn (R.Z.); RWu@bjfu.edu.cn (R.W.)

**Keywords:** DNA-binding site, prediction, machine learning method, bioinformatics

## Abstract

Interactions between proteins and DNA play an important role in many essential biological processes such as DNA replication, transcription, splicing, and repair. The identification of amino acid residues involved in DNA-binding sites is critical for understanding the mechanism of these biological activities. In the last decade, numerous computational approaches have been developed to predict protein DNA-binding sites based on protein sequence and/or structural information, which play an important role in complementing experimental strategies. At this time, approaches can be divided into three categories: sequence-based DNA-binding site prediction, structure-based DNA-binding site prediction, and homology modeling and threading. In this article, we review existing research on computational methods to predict protein DNA-binding sites, which includes data sets, various residue sequence/structural features, machine learning methods for comparison and selection, evaluation methods, performance comparison of different tools, and future directions in protein DNA-binding site prediction. In particular, we detail the meta-analysis of protein DNA-binding sites. We also propose specific implications that are likely to result in novel prediction methods, increased performance, or practical applications.

## 1. Introduction

Protein–DNA interactions are widely distributed in all living organisms. Previous reports have estimated that 2%–3% of a prokaryotic genome and 6%–7% of a eukaryotic genome encodes DNA-binding proteins [1,2]. The interactions can be formed by different domains, such as the zinc finger or the helix-turn-helix. These interactions are involved in a variety of biological processes including DNA replication, DNA repair, viral infection, DNA packing, and DNA modifications [3]. Among them, transcription factors (TFs) are the best characterized. When a TF binds to specific DNA sequences in promoters, they can promote or repress the transcription of genes [4].

Although more than 105,839 experimentally determined structures are deposited in the current (December 2014) Protein Data Bank (PDB) database [5], only several thousand protein–DNA complex structures are listed, which is much smaller than the number of protein–DNA complexes that occur in nature. In recent years, several genome sequences of various organisms have been completed with the development of next-generation sequencing technology. Huge numbers of DNA and protein sequences have been produced, many of which are DNA-binding proteins. Examining how and when protein–DNA interactions occur would increase our understanding of the genome. A full picture of the interactions will allow for characterization of genes transcribed at any given time in response to a dynamic changing environment.

Traditionally, the DNA-binding proteins or residues can be identified using various experimental techniques, such as electrophoretic mobility shift assays (EMSAs) [6,7], conventional chromatin immunoprecipitation (ChIP) [8], MicroChIP [9], Fast ChIP [10], peptide nucleic acid (PNA)-assisted identification of RNA binding proteins (RBPs) (PAIR) [11], X-ray crystallography [12], and nuclear magnetic resonance (NMR) spectroscopy [13]. However, these approaches are time-consuming and expensive. Moreover, with the huge amount of protein sequence data available, developing computational tools that can rapidly and reliably identify DNA–binding proteins or residues is important [14]. Compared to Wet lab experiments, computational methods can rapidly and cheaply identify DNA-binding sites, which are very useful in understanding biological functions. In the last three decades, many efforts have been made to develop more accurate and efficient approaches in this area. These methods have focused on two aspects: determining whether a protein interacts with DNA and predicting the binding sites. These computational methods have become more accurate and are providing large amounts of data.

**Figure 1:**
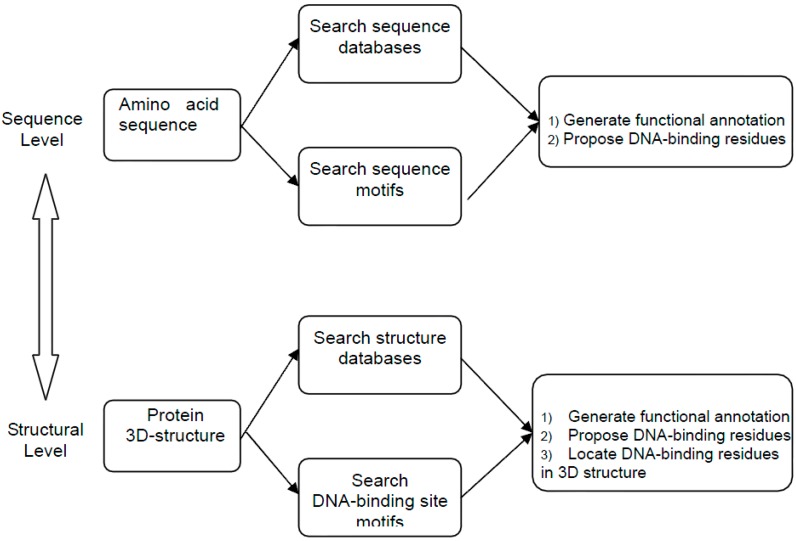
Sequence similarity and structure similarity-based strategies.

Predictions based on sequence and structural information comprise major computational strategies commonly used to identify DNA-binding residues in a query protein. The sequence similarity-based methods require the identification of homologous sequences with known DNA-binding residues. Based on sequence alignment of the query protein and an identified homolog, DNA-binding residues in the query sequence can be inferred from the identified homolog. Several studies have focused on methods for DNA–binding prediction from sequence similarity [3,15,16,17,18,19]. However, such methods cannot typically achieve satisfactory performance because DNA-binding residues are less conserved [20]. When the protein of interest has a known structure, structure-based methods are used to detect DNA-binding residues [20,21,22,23]. The potential DNA-binding sites in the query protein can be predicted by comparing known binding site structures to the query protein. Generally, three-dimensional (3D) structural information can be used to obtain in-depth insights into the binding site. However, a good structural match is not necessarily indicative of a similar function, which has been supported based on other functional sites [24,25]. Sequence similarity and structure similarity-based strategies are shown in Figure 1. Another technique is homology modeling and threading, a method in which a target protein with an unknown structure is modeled by identifying a template protein of known structure. If no structure is available, a 3D model can be generated using homology modeling or threading. The models are not entirely accurate, but can still be used for DNA-binding site prediction [26,27,28]. In addition, this approach can be used as a complementary strategy. Because the known DNA-binding residue sequence/structural features are limited, each prediction method has its own disadvantages. Thus, meta-prediction [29] and comparative studies [30] have been performed, which have analyzed the state-of-the-art prediction methods and achieved higher accuracy and sensitivity to provide diverse and useful prediction tools for protein DNA-binding sites.

In this review, we discuss the essential biological role of protein–DNA interactions and the complete picture of DNA-binding proteins or residues using experimental strategies or computational methods. Although the prediction methods are based on different benchmark data sets or different evaluation criteria, which complicates the comparison of disadvantages and advantages of various methods, we summarize these studies. The main contents of this report include benchmark data sets, various residue sequence/structural features, machine learning method comparison and selection, evaluation methods, and performance comparison of different tools. In particular, we detail the meta-analysis of protein DNA-binding sites. Furthermore, we discuss future directions and some implications that are likely to result in novel prediction methods, increased performance, or practical applications in the topic of protein DNA-binding site prediction.

## 2. Method Development of DNA-Binding Site Prediction

### 2.1. Benchmark Data Set

The sequence and 3D structural data on protein–DNA complexes are available from public databases. Several previous studies have constructed their own benchmark data sets, the details of which are listed in Table 1. In these cases, DBP374 was the largest database, which is optimal for initiating novel studies. If possible, new studies on DNA–protein interactions should employ a data set that has already been used in the literature, which facilitates direct comparison with previous studies. Additionally, two specific databases are devoted to protein–DNA interactions using available information from the PDB [31,32].

**Table 1:** Commonly used data sets for DNA-binding site identification.

ID	Ref. No.	Notes
DB179	[33]	179 DNA-binding proteins, almost entirely nonredundant at 40% sequence identity
NB3797	[33]	3797 nonbinding proteins, significant redundancy at 35% sequence identity level (only 3482 independent clusters)
PD138	[27]	138 DNA-binding proteins, almost entirely nonredundant at 35% sequence identity, divided into seven structural classes
DISIS	[3]	78 DNA-binding proteins, close to nonredundant at 20% sequence identity
PDNA62	[34]	62 DNA-binding proteins, 78 chains, 57 nonredundant sequences at 30% identity.
NB110	[34]	110 nonbinding proteins, nonredundant at 30% sequence identity level, derived from the RS126 secondary structure data set by removing entries related to DNA
BIND54	[35]	Reported as 54 binding proteins, actually 58 chains, nonredundant at 30% sequence identity, original list of proteins was reported in [1]
NB250	[35]	250 nonbinding proteins, mostly nonredundant at a 35% sequence identity
DBP374	[18]	374 DNA-binding proteins, significant redundancy at a 25% sequence identity level
TS75	[18]	75 DNA-binding proteins, designed to be independent from DBP374 and PDNA62 but has some redundant entries in both at a 35% sequence identity level
PDNA-316	[29]	316 target proteins used in metaDBSite Web server, at 30% sequence identity
DNABindR171	[16]	171 proteins with mutual sequence identity ≤30% and each protein has at least 40 amino acid residues. All the structures have resolution better than 3.0 Å and an R factor less than 0.3

In a protein–DNA complex, an amino acid residue in the protein is defined as a binding site if the distance between any atoms of this residue and any atoms of the DNA molecule is less than a specific cutoff value. Several previous studies on DNA–protein binding site prediction have used various definitions of DNA-binding sites [34,36,37]. Kuznetsov *et al*. [38] declared that the cutoff distance of 4.5 Å gave the best separation between binding and nonbinding residues when using evolutionary and structural information to predict binding sites, while Si *et al*. [29] applied cutoff distances of 3.5, 4.0, 4.5, 5.0, 5.5, 6.0 Å, and binding sites with the solvent accessible surface area (ASA) in two data sets and chose 3.5 Å as the most proper definition.

The sequence similarity among proteins in the data set is important to the prediction result. The current methods declare that the similarity level should be kept below 30%–35%. A single representative from each protein cluster was identified and subsequences of other proteins in a data set were eliminated. The frequently used clustering program contains blastclust (available from the National Center for Biotechnology Information, NCBI), the PISCES Web server [39], and the H-CD-HIT program [40]. Using the metaDBSite method, the protein–DNA complexes with a resolution better than 3.0 Å were submitted to H-CD-HIT to obtain a nonredundant data set, which was first clustered at a high identity (90%), after which the nonredundant sequences were further clustered at a low identity (60%). A third cluster was generated at a lower identity (30%). Default clustering parameters were selected in H-CD-HIT.

When using the data set for cross-validation, ensuring that divisions were performed at the DNA-binding residue prediction level is important. Residues from the same protein should not appear in both training and testing sets; residues from binding sites and nonbinding sites were signed as +1 and −1, respectively. When reporting performance, a variety of measures should be applied, especially a receiver operating characteristic (ROC) analysis [41] and the Matthews correlation coefficient (MCC).

### 2.2. Different Residue Properties Used in Developing Predictors

To construct the DNA-binding residue predictor, numerous features have been employed to identify DNA-binding or nonbinding proteins and residues. Generally, the protein features can be classified into sequence-based features, structure-based features, and physical and chemical features, such as hydrophobicity. In recent years, evolutionary information has been used in the prediction system, which has helped achieve a higher prediction rate. Residue properties reviewed here include amino acid sequence/residue type, sequence conservation, evolutionarily conserved residues, the global composition of amino acids, structural motifs, structural neighborhood, structural flexibility, secondary structure, ASAs, hydrophobicity, electrostatic potentials, net charge, and dipole and quadrupole moments. We introduce the physicochemical implications of these residue properties and review necessary bioinformatic tools to obtain the encoding information, which can be used as a basis for future studies. More details on these residue features are described below.

#### 2.2.1. Sequence-Based Features

**Amino acid sequence**/**residue type:** Protein amino acids are the most common feature of any sequence-based predictor, which also applies to DNA-binding site predictors. The protein’s amino acid sequence provides basic information to the predictor.

Twenty amino acids have different propensities, such as charged residues (H, R, K, E, D), polar residues (Q, T, S, N, C, Y, W), and hydrophobic residues (G, F, L, M, A, I, P, V) [42], with two frequently used encodings used to represent the properties of different residue types. The first is the standard binary encoding, which details each amino acid encoded as a 20-dimensional binary vector, such as A (1 0 0 0 0 0 0 0 0 0 0 0 0 0 0 0 0 0 0 0 0), C (0 1 0 0 0 0 0 0 0 0 0 0 0 0 0 0 0 0 0 0), D (0 0 1 0 0 0 0 0 0 0 0 0 0 0 0 0 0 0 0 0), E (0 0 0 1 0 0 0 0 0 0 0 0 0 0 0 0 0 0 0 0), …, and Y (0 0 0 0 0 0 0 0 0 0 0 0 0 0 0 0 0 0 0 1). The secondary encoding is according to different residue types based on physicochemical properties. For example, the three different amino acid types (charged, polar, and hydrophobic residues) can be encoded as (0 0), (0 1), and (1 0), respectively [43].

In particular, positively charged residues such as arginine are more likely to interact with the negatively charged backbone of DNA and these positively charged residues have been analyzed and used in previous studies [44].

**Sequence conservation:** The encoding of sequence conservation, also called sequence profiles, is one of the most important features used in the DNA-binding site predictors. Sequence profiles rather than raw sequences are generally believed to provide important information for the prediction system. Profiles are typically generated using iterated PSI-BLAST searches [45]. Generally, the query protein sequence is analyzed against the NCBI nonredundant protein sequence database to obtain a multiple sequence alignment (MSA). The MSA is then used to calculate the sequence conservation score for each residue. A series of conservation scoring tools is available, such as Shannon entropy, von Neumann entropy [46], and relative entropy. In addition, several user-friendly Web servers are commonly used, such as the Scorecons server (Available online: http://www.ebi.ac.uk/thornton-srv/databases/cgi-bin/valdar/scorecons_server.pl). In each residue position, a positive number indicates a favorable substitution and a negative number indicates an unfavorable substitution.

**Evolutionarily conserved residues:** Evolutionary data have been developed based on sequence conservation information through phylogenetic analyses. Recently, evolutionary information has been used in a series of functional site predictions, especially in DNA-binding site prediction. Previous studies have shown that evolutionary information is a powerful feature in distinguishing DNA–binding or nonbinding sites [47,48]. Wang *et al*. [49] proposed a novel method to measure evolutionary conservation, named the state to step ratio score (SSR). In this approach, SSR values for each residue are calculated using variation patterns from the root of the tree (theoretical ancestral sequence) to the leaf of the tree (sequences in MSA). The tree here was constructed based on the MSA (calculated using PSI-BLAST). The SSR score has been considered an effective evaluation tool for measuring evolutionary conservation and has been defined as a powerful descriptor for distinguishing DNA-binding and nonbinding residues. However, regardless of how the evolutionary conservation information was obtained (using sequences or combined with structural information), a sufficient number of diverse homologs are available.

**Global composition of amino acids:** Due to the DNA-binding protein or residue identification, the counts or frequencies of each type of amino acid are often used, and pairs of adjacent residues or three neighboring residues or residue windows are applied as compositional features [50]. The encoding methods here are similar to the residue type. To represent pairs of adjacent residues, two columns of 20-dimensional vectors are needed. In addition, three columns of 20-dimensional vectors are required for three neighboring residues.

#### 2.2.2. Structural-Based Features

**Structural motifs:** Many known DNA-binding protein structures have certain distinct structural motifs that act in the binding site [51]. Shanahan *et al*. [52] developed a classic method to identify DNA-binding proteins mainly using structural motifs. The authors focused on three important motifs, namely, the helix-turn-helix (HTH), the helix-hairpin-helix (HhH), and the helix-loop-helix (HLH) motifs. Based on the names, all three motifs start and terminate with helices, connected by a short linking region of varying geometry. These motif structures are always considered to be flexile and more likely to form functional sites. Jones *et al*. found that the HTH motif accounts for a significant number of all DNA-binding proteins [7]. Winged-helix was one of rather common motifs in transcription factors, which represents a sub-class of the HTH motif. Identifying such motifs in a novel protein can help to distinguish DNA-binding proteins from other proteins.

**Structural neighborhood:** The spatial neighbors of a residue can accurately represent the residue’s environment. Kuznetsov *et al*. [38] defined spatial neighbors of a residue as a vector of dimension 20 that contains the normalized frequencies of occurrence of the 20 amino acid types. The position of each residue is described using the coordinates of its *C*^α^ atom. This count number of other amino acids around the residue of interest is believed to be an important structural descriptor to predict DNA-binding sites.

**Structural flexibility:** To accommodate bound DNA, the binding residues in proteins tend to be more flexible than other residues [53], a common characteristic in a series of functional sites [42,54]. Previous studies have shown that the encodings based on the B-factors are frequently used to measure residue flexibility [55,56]. Note that this encoding may only be suitable for structures determined using X-ray crystallography.

**Secondary structure:** The secondary structure state (SSS) represents the local, repeated geometric patterns in proteins. For sequence-based prediction, the SSS of a residue can be predicted via secondary structure prediction methods [57,58] (e.g., PSIPRED [53]); for a structural-based prediction, the SSS can be calculated from the corresponding structure through secondary structure assignment methods [59] (e.g., DSSPcont [60]). Several studies have employed the SSS of a residue as the encoding information in the prediction of DNA–binding residues. Note that the secondary structure was not considered a particularly informative feature to identify DNA-binding sites in previous reports [34,44].

**Accessible surface area (ASA):** DNA-binding residues tend to be exposed to a solvent to form contacts with the DNA structure, which makes relative solvent accessibility a useful predictive feature. Some studies have focused only on surface residues in the prediction [61]. Similar to the secondary structure, the relative ASA can be predicted based on the protein sequence or calculated from the protein structure using specific software (e.g., NACCESS [62]). The relative ASA of each residue in a protein was calculated when the DNA molecule was present (non-complexed). Non-complexed was considered to be the protein structure extracted alone from the PDB file. Surface accessible residues were defined as residues with a relative ASA of >5%. As reported in the literature [54], five ASA-based encodings were explored, including the ASA of all atoms (*Aa*A*SA*), the ASA of all side chain atoms including alpha carbons (*As*A*SA*), the residue accessibility (RSA) of nonpolar side chain atoms (*Np*A*SA*), the RSA of all polar side chain atoms (*Ap*A*SA*), and the RSA of all main chain atoms (*Mc*A*SA*).

#### 2.2.3. Physical and Chemical Features

**Hydrophobicity:** Hydrophobicity is the physical property of a molecule that is repelled by water. Faucher and Pliska [63] applied experimentally derived amino acid hydrophobicity to assign the value to each surface patch. Kate and Doolittle [64] assigned a hydrophobicity scale with a fixed numerical value to each of the 20 amino acids. The hydrophobicity has been commonly used in DNA-binding predictors.

**Electrostatic potentials:** The computed electrostatic properties were also explored to predict DNA-binding residues. Molecular dynamics software is used to compute the charges for each atom, which is usually averaged to assign an electrostatic score to each residue [35,36,52,65,66]. Software is also available for the calculation [66]. Susan Jones *et al*. developed a method for DNA-binding site prediction using electrostatic potentials. In their work [36], the electrostatic potential was computed for individual protein chains (without DNA) using the software package Delphi. The potential was computed on a discrete cubic grid, with points in the *x*, *y*, and *z* directions, which determined that the protein filled 30% of the total volume. Debye-Hückel boundary conditions were used (the default for this package), as well as a simplified charge set defined from the molecular dynamics package CHARMM [67].

**Net charge, and dipole and quadrupole moments:** The net charge, electric dipole moment, and quadrupole moment measure how widely an electric charge is distributed across the protein. Ahmad and Sarai previously showed that the magnitudes of the moments of electric charge distribution in DNA-binding sites are significantly different from those in nonbinding sites [68]. Net charge, and dipole and quadrupole moments, could respectively distinguish binding and nonbinding proteins with reasonable performance. The combinations of these features make a fairly discriminatory feature in DNA-binding protein prediction.

### 2.3. Prediction Methods

In existing studies, the predictors of DNA-binding residues or proteins can be divided into three approaches according to known information: only the protein sequence is known; the structure of the query protein has been resolved; and the query protein’s structure was unknown but could be modeled by identifying a template protein of known structure. The three methods based on different information are detailed below.

#### 2.3.1. Prediction Based on Sequences

For initial DNA-binding protein prediction studies, the methods typically utilized only sequence residues because the protein structural information was limited. Such methods distinguish whether a protein binds DNA or which residues bind DNA without structural contributions. Generally, the sequence similarity was found by searching the NCBI nonredundant protein sequence database using BLAST or PSI-BLAST. Several other sequence-based features can be used to recognize DNA-binding sites, such as the amino acid sequence, residue type, sequence conservation, evolutionarily conserved residues, and global composition of amino acids. Representing single-residue sequence information to the machine learning is relatively simple, and several studies have focused on sequence-based predictions [3,15,16,17,18,19].

Over time, numerous DNA-binding protein structures have been obtained using experimental methods, so the data sets used in the prediction work have expanded. In this case, methods based on sequences alone cannot achieve satisfactory performance because DNA-binding residues are less conserved [20]. Thus, adding available structural information is important to improve prediction.

#### 2.3.2. Prediction Based on Protein Structures

Without a structural template, the predictors usually provide inferior performance. DNA-binding site predictions will likely be improved by employing available structural information [28]. The protein structures can be determined based on several methods, such as X-ray crystallography and NMR spectroscopy. Structure information could be obtained from The PDB database, which is the professional resource of 3D structures of proteins, nucleic acids, and complex assemblies. DNA-binding proteins typically have two available structures: the bound complex when DNA is present (holo protein conformation) and the unbound protein when DNA is absent (apo protein conformation).

The potential DNA-binding sites in the query protein can be inferred as those structurally aligned to known binding sites. Generally, the 3D structural information can be used to obtain in-depth information on the binding site. However, the presence of a good structural match is not necessarily indicative of a similar function, an idea supported based on other functional sites [24,25]. Rather than directly relying on structural similarity to identify DNA-binding sites, the descriptors (e.g., structural motifs and electrostatics) exploited from structural information have been employed in the machine learning process to predict these sites [20,21,22,23].

#### 2.3.3. Homology Modeling and Threading

Another technique different from traditional sequence-based and structure-based methods is homology modeling and threading. In both techniques, a target protein with an unknown structure can be modeled by identifying a template protein of known structure. If no query structure is available, its 3D model can be generated using homology modeling or threading [69]. Identifying a fit template with a known structure is important, which is usually achieved based on a combination of sequence similarity and energy calculations.

When the target protein structure is unknown, homology modeling or threading may be able to model it. In most cases, homology models are not entirely accurate, but can be used to determine whether the protein binds DNA [26,27,28]. Note that template-based methods are not always successful. Over 40% of DNA-binding proteins have no suitable template for homology modeling according to previous studies [3]. If no suitable template is available, this technique cannot be used to predict DNA-binding site, although it can be applied as a complementary strategy for DNA-binding site prediction.

### 2.4. Prediction Algorithms

#### 2.4.1. Prediction Algorithms Based on Individual Descriptors 

In the early studies of DNA-binding site prediction, many residue properties have been employed to identify DNA-binding residues. Several algorithms based on the residue properties have been developed [7,36,52,65]. Some of the predictors rely on individual descriptors. For example, the empirical preference of electrostatic potential and the shape of molecular surfaces were used to predict DNA-binding sites [65]. Jones *et al*. [36] developed a DNA-binding site predictor using electrostatic potential information. Similarly, Shanahan *et al*. [52] explored a method using structural motifs and the electrostatic potential. The advantage of this method is the simplicity, but the disadvantage is the weak predictive performance due to the limited number of descriptors.

#### 2.4.2. Prediction Algorithms Based on Simple Statistical Methods

To improve the accuracy of the predictors, combining the different descriptors and using powerful statistical methods and state-of-the-art machine learning methods is essential. Independent descriptors can provide complementary information to improve the predictor’s performance. Szilagyi and Skolnick [27] developed an efficient method using a linear formula, with coefficients derived from logistic regression. The descriptors used in this method were the relative proportions of certain amino acids in the protein sequence, the asymmetry of the spatial distribution of certain other amino acids, and the dipole moment of the molecule. The simple statistical model was effective in distinguishing protein functional sites, including DNA-binding sites [70].

#### 2.4.3. Prediction Algorithms Based on Machine Learning Methods

In past decades, the majority of state-of-the-art machine learning methods have been applied to DNA-binding protein and DNA-binding residue prediction. In a classification system, a classifier approximates a function from the training data and attempts to identify the correct output from a given unknown feature vector[71]. Generally, the machine learning methods could obtain better performance than the preceding two methods. Here, we introduce several frequently used machine learning methods including support vector machines (SVMs) [3,72], artificial neural networks (ANN) [35,61], decision tree [19], Bayesian network [16], and random forest [18,48].

**Support vector machines (SVMs):** As a machine-learning method for two classes of classification, SVMs aim to identify a rule that correctly puts each member of a training set into the corresponding class. Using the kernel function, the SVMs could resolve nonlinear problems [73]. The SVM algorithm has been widely applied in biological areas, including the prediction of protein structures and functions, as well as the prediction of DNA–protein interactions due to its nonlinearities and high dimensional characteristics [56,74,75,76,77]. The public and popular SVM algorithms include SVM^light^ and LIBSVM. The SVM^light^ was encoded by C/C++ and is available at http://svmlight.joachims.org/. LIBSVM is encoded by C/C++ and matlab, and is available at http://www.csie.ntu.edu.tw/cjlin/libsvm. For DNA-binding site prediction, SVM is used to distinguish DNA-binding residues from nonbinding residues. DNA-binding amino acids are considered positive samples and non-DNA-binding amino acids are considered negative samples.

**Artificial neural networks (ANN):** Artificial neural networks (ANN) are another commonly used method in protein functional site prediction [35,61]. The neural networks approach has several advantages such as the ability to perform multiple training steps, the capability of detecting all possible interactions, and the fact that it requires less formal statistical training. When using the ANN algorithm, more structure-based descriptors are involved as input features. The computational complexity depends on the dimensionality of input vectors [78]. The prediction process started with random weight training and subsequently optimized feature weights by comparing the calculated results with the expected results [34]. The available ANN software packages can be downloaded from Open NN (Available online: http://www.cimne.com/flood/download.asp).

**Decision tree:** A decision tree is a decision support tool that uses a treelike model of decisions and their possible consequences [19]. A decision tree is simple to understand and interpret, but its prediction sensitivity is influenced by the quality and complexity of input data. Biological data are large and complex and the decision tree cannot always meet the needs of prediction problems in bioinformatics. In recent years, several modified methods based on decision trees were developed, such as random forest, boosted decision trees, and alternating decision trees. These methods are known to be more powerful for DNA-binding site prediction [18,79,80]. The available decision tree software includes C4.5 (Available online: http://www2.cs.uregina.ca/~dbd/cs831/notes/ml/dtrees/c4.5/tutorial.html) and spider (Available online: http://www.kyb.mpg.de/bs/people/spider/).

**Random forest:** Random forest is an ensemble classifier that consists of multiple decision trees with controlled variations [81]. The primary concept of the algorithm is that the general model is decided by training multiple decision trees, and that the multiple decision trees are used for classification. Only two parameters are required in the random forest algorithm, e.g., the number of decision trees and the number of input features split in each tree. The random forest method has already been used in DNA-binding site prediction [18,48]. The software can be downloaded from Random Forests (Available online: http://www.stat.berkeley.edu/~breiman/RandomForests/cc_home.htm).

**Bayesian learning:** Bayesian learning methods were developed based on the Bayesian theorem. In the Bayesian classification, the probability of the given and unknown input in various categories is calculated, and the unclassified item is considered to belong to the category with the largest probability. Bayesian learning methods are effective when the input information is high dimensional [82,83]. The naive Bayesian classifier is a Bayesian model that has been successfully applied for predicting DNA-binding sites in the Weka package (Available online: http://www.cs.waikato.ac.nz/ml/weka/) [16]. Another Bayesian learning software, bnt, is available at http://code.google.com/p/bnt/.

#### 2.4.4. Hybrid Learning and Meta-Prediction Methods

Because the known DNA-binding residues sequence/structure features are limited, each prediction method has its own disadvantages. Thus, meta-prediction [29] and comparative study work [30] is required. These methods are robust and effective in many applications, including DNA-binding site prediction. For example, the prediction method DISPLAR was constructed using two-layer neural networks [61], and SeqPredNet was constructed using a delicate three-layered network [34]. The metaDBSite integrated six online Web servers to predict and analyze DNA-binding sites, and showed higher accuracy and sensitivity [29]. These studies provide diverse and useful prediction tools for protein DNA-binding sites.

### 2.5. Performance and Evaluation of Different Predictors

#### 2.5.1. Performance Measures

As reported previously, we present several commonly used measures of prediction performance, *i.e.*, accuracy, sensitivity, specificity, strength, the MCC, precision, F-measure, and area under the ROC curve (AUC), which are listed in Table 2.

**Table 2:** Evaluation parameters.

Parameter	Meaning	Expression
Accuracy (ACC)	Percentage of correct prediction	$Accuracy=\frac{TP+TN}{TP+TN+FP+FN}$ ^a^
Sensitivity	Percentage of correctly predicted positive	$Sensitivity=\frac{TP}{TP+FN}$
Specificity	Percentage of correctly predicted negative	$Specificity=\frac{TN}{TN+FP}$
Strength	Mean value of the sum of sensitivity and specificity	$Strength=\frac{Sensitivity+Specificity}{2}$
MCC	Matthews correlation coefficient	$MCC=\frac{\left(TP\times TN)-(FN\times FP\right)}{\sqrt{\left(TP+FN)\times (TN+FP)\times (TP+FP)\times (TN+FN\right)}}$
Precision	Positive predictive rate	$Precision=\frac{TP}{TP+FP}$
F-measure	The harmonic mean of sensitivity and specificity	$F-measure=\frac{2\times Presion\times Sensitivity}{Presion+Sensitivity}$
AUC ^b^	Probability that a classifier will rank a randomly chosen positive instance higher than a randomly chosen negative one	$AUC=\frac{\sum_{i=1}^{n}T_{i}}{nT}$

^a^: TP = True positive number; TN = True negative number; FP = False positive number; FP = False negative number; ^b^: In AUC formulation, *i* takes on values from 1 to *n*, *T* is the total number of positives in the test set, and *Ti* is the number of positives that score higher than the *i* th highest scoring negative.

The MCC, which measures the quality of classifiers, was considered suitable for evaluating the overall prediction accuracy due to the disparate number of DNA-binding sites and nonbinding sites in real protein–DNA complexes. An MCC of 1 represents a perfect prediction, whereas an MCC of 0 indicates a completely random prediction. The ROC curve is another widely used measurement to evaluate prediction performance, especially in multiple predictor evaluation. A ROC curve plots the false positive rate against the true positive rate. A perfect predictor would have an AUC of 1, whereas a classifier that makes random guesses would have an AUC of 0.5.

Regarding the formulation in Table 2, note that TP represents true positives (residues predicted to be DNA-binding residues that are in fact not interface residues), TN indicates true negatives (residues predicted to be non-DNA-binding residues that are in fact not DNA-binding residues), FP denotes false positives (residues predicted to be DNA-binding residues that are in fact not interface residues), and FN represents false negatives (residues predicted to be non-DNA-binding residues that are in fact DNA-binding residues).

#### 2.5.2. Comparison of Different Prediction Methods

Table 3 summarizes the performance of the state-of-the-art methods for DNA-binding site prediction. The accuracy of most predictors was around 70%–90%, and the sensitivity and specificity of these methods are about 70%–80%. Ofran *et al*. [3] got high score acc = 0.890. In contrast, another dataset PDNA-316 was used in Ofran’s method, the high scores acc = 0.920 and spe = 0.980 but a low score sen = 0.190 were obtained [29]. Ozbek has got similar results, with excellent acc and spe but low sensitivity [84]. These predict results were often happened because of the great disparity between positive samples (DNA-binding sites) and negative samples (non DNA-binding sites). Different methods have their own advantages and disadvantages due to their own data set usage and different prediction algorithm usage. In particularly, the small size of dataset makes the predictor unstable and dubitable. The MCC is known to be a proper and unbiased measurement to evaluate overall performance, but the majority of these methods did not calculate this value. Subsequently, we developed the metaDBSite method, which combined several predictors and used the same PDNA-316 data set to measure its overall performance. The results of metaDBSite, detailed in Table 3 italic lins, show more reliable prediction results.

#### 2.5.3. Selected Web Servers of DNA-Binding Site Predictors

At this time, several DNA-binding sites prediction Web servers are available, and these URLs are summarized in Table 4. These methods contain sequence-based and structure-based predictors. Machine learning methods are applied in most of these predictors. To develop a bioinformatics tool, providing a Web server is important to the community at large as well as to developers. Free and easy-to-use Web servers can help users experience the power of these algorithms and then maximize their applications. Meanwhile, feedback from users will urge developers to continuously improve their algorithms. Focusing on DNA-binding site prediction, these Web servers help experimental scientists accelerate the functional characterization of protein–DNA complexes. By learning from the protein fold recognition community [85] and protein-protein interaction meta-server [86], a meta-server for DNA-binding site prediction has been developed [29]. Users can take advantage of results from different predictors to obtain more reliable predictions.

**Table 3:** Performance of the state-of-the-art methods for DNA-binding site prediction.

Author & Year	Data set (own/PDNA-316)	Performance								Alogrithm ^b^	Reference
		ACC	SEN	SPE	AUC	MCC	Strength	F-Measure	Precision		
Jones 2003	own	0.680								1	[36]
Ahmad 2004	own	0.664	0.682	0.660						2	[34]
^a^ *Ahmad 2004*	*PDNA-316*	*0.750*	*0.530*	*0.760*		*0.170*	*0.650*	*0.230*		*3*	[29]
Ferrer-Costa 2005	own	0.835								4	[87]
Kuznetsov 2006	own	0.760	0.769	0.747	0.830	0.450				3	[38]
Wang 2006	own	0.703	0.694	0.704	0.750					3	[88]
*Wang 2006*	*PDNA-316*	*0.780*	*0.540*	*0.800*		*0.210*	*0.670*	*0.260*		*3*	[29]
Yan 2006	own	0.710	0.530	0.350						5	[16]
*Yan 2006*	*PDNA-316*	*0.730*	*0.660*	*0.740*		*0.230*	*0.700*	*0.260*		*3*	[29]
Tjong 2007	own	0.680								2	[61]
Ofran 2007	own	0.890								2/3	[3]
*Ofran 2007*	*PDNA-316*	*0.920*	*0.190*	*0.980*		*0.250*	*0.590*	*0.270*		*3*	[29]
Hwang 2007	own	0.772	0.764	0.766						3	[15]
*Hwang 2007*	*PDNA-316*	*0.780*	*0.690*	*0.790*		*0.290*	*0.740*	*0.310*		*3*	[29]
Nimrod 2009	own		0.900		0.900				0.350	6	[48,89]
Wang 2009	own	0.800	0.731	0.806	0.850					6	[80]
*Wang 2009*	*PDNA-316*	*0.820*	*0.670*	*0.830*		*0.320*	*0.750*	*0.340*		*3*	[29]
Wu 2009	own	0.914	0.766	0.944						6	[18]
Carson 2010	own	0.785	0.797	0.772	0.860	0.570				7	[19]
Ozbek 2010	own	0.960	0.360	0.990						8	[84]
*Si 2011*	*PDNA-316*	*0.770*	*0.770*	*0.770*		*0.320*	*0.770*	*0.330*		*3*	[29]

^a^: Italic lines represent the performance of previous methods using the PDNA-316 data set (from the metaDBSite method); ^b^: Patch prediction = 1, Neural Network = 2, SVM = 3, Linear Predictor = 4, Naïve Bayes = 5, Random Forest = 6, C4.5BAGCST = 7, Gaussian Network Model = 8.

**Table 4:** A selection of DNA-binding protein or residues prediction Web servers.

Methods	URLs	References	Publication Year
newDNA-Prot	http://sourceforge.net/projects/newdnaprot/	[90]	2014
DNABind	http://mleg.cse.sc.edu/DNABind/	[91]	2013
DNABR	http://www.cbi.seu.edu.cn/DNABR/	[92]	2012
DR_bind	http://dnasite.limlab.ibms.sinica.edu.tw	[93]	2012
MetaDBSite	http://projects.biotec.tu-dresden.de/metadbsite/ http://sysbio.zju.edu.cn/metadbsite	[29]	2011
DNABINDPROT	http://www.prc.boun.edu.tr/appserv/prc/dnabindprot/	[84]	2010
bindn-rf	http://bioinfo.ggc.org/bindn-rf/	[80]	2009
DBindR	http://www.cbi.seu.edu.cn/DBindR/DBindR.htm	[18]	2009
DP-Bind	http://lcg.rit.albany.edu/dp-bind	[15,38]	2007
BindN	http://bioinfo.ggc.org/bindn/	[88]	2006

### 2.6. Status of the Prediction of Protein-Binding Sites in DNA Sequence

As reported previously, the prediction of the interaction between DNA and proteins focused on two aspects: DNA-binding sites prediction and protein (especially transcription factor)-binding sites prediction. In this review, we mainly introduce the DNA-binding sites prediction. Although fewer researchers work on the development of DNA-centered methods, it is still necessary to discuss the approaches for prediction of protein-binding sites focusing on the DNA sequence.

As the representative of protein-binding sites, transcription factor binding sites (TFBS) were well studied. Several features contributed to TFBS recognition, including the nucleotide sequence, 3D structure of protein and binding residues, cooperative DNA-binding of TFs, chromatin accessibility, nucleosome occupancy, DNA methylation, *etc.* [94]. Similar algorithms (e.g., PSI-BLAST, SVM, neural networks, *etc.*) have been used in TFBS prediction. The performance and evaluation in TFBS prediction were the same as predicting DNA-binding sites on the protein sequence. The pivotal descriptor was the shape information of protein-binding sites. Yang *et al*. [95] have developed a TFBSshape database which could be used to calculate DNA structural features and provide mechanistic insights into protein–DNA recognition and help to improve the prediction accuracy.

## 3. Future Perspectives

In conclusion, the prediction of DNA-binding sites is an increasingly important topic in the field of protein functional site prediction, and visible improvements have occurred in the past several years. The current available Web servers play an important role in helping experimental scientists accelerate the functional characterization of protein–DNA complexes. However, the overall performances of various prediction methods remain low, and the predictors could be more effective in practical use.

To improve the predictive performance of DNA-binding sites, the following aspects can be addressed. First, due to the protein and DNA complex, the binding site information relies on both the protein and DNA structure. The current predictors apply machine learning methods that consider various characteristics of protein and DNA, but do not consider conformational changes during the process of DNA-binding to the protein. The molecular dynamics and ligand docking methods, which are commonly used in several studies of protein interactions with small molecules, may also be employed for DNA-binding proteins [96,97]. Second, several studies have developed models for TFs, which are the most important DNA-binding proteins [74,98,99,100,101]. Both DNA and protein information have been used to train classifiers for TFs. The specific classifier for each TF family could be developed directly according to the different features of each TF family, which could yield higher prediction accuracy [101]. Third, many features are typically used by the DNA-binding site predictors. Combining different feature information is useful for correctly identifying DNA-binding residues, but the high-dimensional space can cause an over-fitting problem [102]. In addition, whether these features contribute to the final prediction and which predictors are more sensitive remain unclear. We believe that the effective feature selection and novel feature mining should be one of the most important approaches in DNA-binding site identification. Fourth, the combination and comparison of various methods have been attempted in previous studies [29,30]. However, further studies are required (e.g., comparison between sequence-based and structure-based predictions to evaluate how much inference capability is gained when the structure is available) to guide further development. Through these efforts, we expect that the overall performance for the prediction of DNA-binding sites could be improved. In addition, we can increase our understanding of the molecular mechanisms of protein–DNA interactions.

## References

[1] Luscombe N.M., Austin S.E., Berman H.M., Thornton J.M. (2000). An overview of the structures of protein–DNA complexes. Genome Biol..

[2] Walter M.C., Rattei T., Arnold R., Guldener U., Munsterkotter M., Nenova K., Kastenmuller G., Tischler P., Wolling A., Volz A. (2009). PEDANT covers all complete RefSeq genomes. Nucleic Acids Res..

[3] Ofran Y., Mysore V., Rost B. (2007). Prediction of DNA-binding residues from sequence. Bioinformatics.

[4] Ptashne M. (2005). Regulation of transcription: From lambda to eukaryotes. Trends Biochem. Sci..

[5] Berman H.M., Westbrook J., Feng Z., Gilliland G., Bhat T.N., Weissig H., Shindyalov I.N., Bourne P.E. (2000). The protein data bank. Nucleic Acids Res..

[6] Jones S., van Heyningen P., Berman H.M., Thornton J.M. (1999). protein–DNA interactions: A structural analysis. J. Mol. Biol..

[7] Jones S., Barker J.A., Nobeli I., Thornton J.M. (2003). Using structural motif templates to identify proteins with DNA binding function. Nucleic Acids Res..

[8] Kono H., Sarai A. (1999). Structure-based prediction of DNA target sites by regulatory proteins. Proteins.

[9] Luscombe N.M., Laskowski R.A., Thornton J.M. (2001). Amino acid-base interactions: A three-dimensional analysis of protein–DNA interactions at an atomic level. Nucleic Acids Res..

[10] Mandel-Gutfreund Y., Margalit H. (1998). Quantitative parameters for amino acid-base interaction: Implications for prediction of protein–DNA binding sites. Nucleic Acids Res..

[11] Olson W.K., Gorin A.A., Lu X.J., Hock L.M., Zhurkin V.B. (1998). DNA sequence-dependent deformability deduced from protein–DNA crystal complexes. Proc. Natl. Acad. Sci. USA.

[12] Orengo C.A., Michie A.D., Jones S., Jones D.T., Swindells M.B., Thornton J.M. (1997). CATH—A hierarchic classification of protein domain structures. Structure.

[13] Ponting C.P., Schultz J., Milpetz F., Bork P. (1999). SMART: Identification and annotation of domains from signalling and extracellular protein sequences. Nucleic Acids Res..

[14] Stormo G.D. (2000). DNA binding sites: Representation and discovery. Bioinformatics.

[15] Hwang S., Gou Z., Kuznetsov I.B. (2007). DP-Bind: A web server for sequence-based prediction of DNA-binding residues in DNA-binding proteins. Bioinformatics.

[16] Yan C., Terribilini M., Wu F., Jernigan R.L., Dobbs D., Honavar V. (2006). Predicting DNA-binding sites of proteins from amino acid sequence. BMC Bioinform..

[17] Ahmad S., Sarai A. (2005). PSSM-based prediction of DNA binding sites in proteins. BMC Bioinform..

[18] Wu J., Liu H., Duan X., Ding Y., Wu H., Bai Y., Sun X. (2009). Prediction of DNA-binding residues in proteins from amino acid sequences using a random forest model with a hybrid feature. Bioinformatics.

[19] Carson M.B., Langlois R., Lu H. (2010). NAPS: A residue-level nucleic acid-binding prediction server. Nucleic Acids Res..

[20] Alibes A., Serrano L., Nadra A.D. (2010). Structure-based DNA-binding prediction and design. Methods Mol. Biol..

[21] Li B.Q., Feng K.Y., Ding J., Cai Y.D. (2014). Predicting DNA-binding sites of proteins based on sequential and 3D structural information. Mol. Genet. Genomics.

[22] Li T., Li Q.Z., Liu S., Fan G.L., Zuo Y.C., Peng Y. (2013). PreDNA: Accurate prediction of DNA-binding sites in proteins by integrating sequence and geometric structure information. Bioinformatics.

[23] Xiong Y., Xia J., Zhang W., Liu J. (2011). Exploiting a reduced set of weighted average features to improve prediction of DNA-binding residues from 3D structures. PLoS One.

[24] Zhang Z., Tang Y., Sheng Z., Zhao D. (2009). An overview of the De Novo prediction of enzyme catalytic residues. Curr. Bioinform..

[25] Nagano N., Orengo C.A., Thornton J.M. (2002). One fold with many functions: the evolutionary relationships between TIM barrel families based on their sequences, structures and functions. J. Mol. Biol..

[26] Morozov A.V., Havranek J.J., Baker D., Siggia E.D. (2005). protein–DNA binding specificity predictions with structural models. Nucleic Acids Res..

[27] Szilagyi A., Skolnick J. (2006). Efficient prediction of nucleic acid binding function from low-resolution protein structures. J. Mol. Biol..

[28] Gao M., Skolnick J. (2009). A threading-based method for the prediction of DNA-binding proteins with application to the human genome. PLoS Comput. Biol..

[29] Si J., Zhang Z., Lin B., Schroeder M., Huang B. (2011). MetaDBSite: A meta approach to improve protein DNA-binding sites prediction. BMC Syst. Biol..

[30] Zhou Q., Liu J.S. (2008). Extracting sequence features to predict protein–DNA interactions: A comparative study. Nucleic Acids Res..

[31] Contreras-Moreira B. (2010). 3D-footprint: A database for the structural analysis of protein–DNA complexes. Nucleic Acids Res..

[32] Norambuena T., Melo F. (2010). The protein–DNA Interface database. BMC Bioinform..

[33] Gao M., Skolnick J. (2008). DBD-Hunter: A knowledge-based method for the prediction of DNA–protein interactions. Nucleic Acids Res..

[34] Ahmad S., Gromiha M.M., Sarai A. (2004). Analysis and prediction of DNA-binding proteins and their binding residues based on composition, sequence and structural information. Bioinformatics.

[35] Stawiski E.W., Gregoret L.M., Mandel-Gutfreund Y. (2003). Annotating nucleic acid-binding function based on protein structure. J. Mol. Biol..

[36] Jones S., Shanahan H.P., Berman H.M., Thornton J.M. (2003). Using electrostatic potentials to predict DNA-binding sites on DNA-binding proteins. Nucleic Acids Res..

[37] Tsuchiya Y., Kinoshita K., Nakamura H. (2005). PreDs: A server for predicting dsDNA-binding site on protein molecular surfaces. Bioinformatics.

[38] Kuznetsov I.B., Gou Z., Li R., Hwang S. (2006). Using evolutionary and structural information to predict DNA-binding sites on DNA-binding proteins. Proteins.

[39] Wang G., Dunbrack R.L. (2005). PISCES: Recent improvements to a PDB sequence culling server. Nucleic Acids Res..

[40] Li W., Godzik A. (2006). Cd-hit: A fast program for clustering and comparing large sets of protein or nucleotide sequences. Bioinformatics.

[41] Linden A. (2006). Measuring diagnostic and predictive accuracy in disease management: An introduction to receiver operating characteristic (ROC) analysis. J. Eval. Clin. Pract..

[42] Bartlett G.J., Porter C.T., Borkakoti N., Thornton J.M. (2002). Analysis of catalytic residues in enzyme active sites. J. Mol. Biol..

[43] Petrova N.V., Wu C.H. (2006). Prediction of catalytic residues using Support Vector Machine with selected protein sequence and structural properties. BMC Bioinform..

[44] Kauffman C., Karypis G. (2008). An analysis of information content present in protein–DNA interactions. Pac. Symp. Biocomput..

[45] Altschul S.F., Madden T.L., Schaffer A.A., Zhang J., Zhang Z., Miller W., Lipman D.J. (1997). Gapped BLAST and PSI-BLAST: A new generation of protein database search programs. Nucleic Acids Res..

[46] Valdar W.S. (2002). Scoring residue conservation. Proteins.

[47] Ahmad S., Keskin O., Sarai A., Nussinov R. (2008). protein–DNA interactions: Structural, thermodynamic and clustering patterns of conserved residues in DNA-binding proteins. Nucleic Acids Res..

[48] Nimrod G., Szilagyi A., Leslie C., Ben-Tal N. (2009). Identification of DNA-binding proteins using structural, electrostatic and evolutionary features. J. Mol. Biol..

[49] Wang K., Horst J.A., Cheng G., Nickle D.C., Samudrala R. (2008). Protein meta-functional signatures from combining sequence, structure, evolution, and amino acid property information. PLoS Comput. Biol..

[50] Kumar M., Gromiha M.M., Raghava G.P. (2007). Identification of DNA-binding proteins using support vector machines and evolutionary profiles. BMC Bioinform..

[51] Harrison S.C. (1991). A structural taxonomy of DNA-binding domains. Nature.

[52] Shanahan H.P., Garcia M.A., Jones S., Thornton J.M. (2004). Identifying DNA-binding proteins using structural motifs and the electrostatic potential. Nucleic Acids Res..

[53] Jones D.T. (1999). Protein secondary structure prediction based on position-specific scoring matrices. J. Mol. Biol..

[54] Yuan Z., Zhao J., Wang Z.X. (2003). Flexibility analysis of enzyme active sites by crystallographic temperature factors. Protein Eng..

[55] Gutteridge A., Bartlett G.J., Thornton J.M. (2003). Using a neural network and spatial clustering to predict the location of active sites in enzymes. J. Mol. Biol..

[56] Tang Y.R., Sheng Z.Y., Chen Y.Z., Zhang Z. (2008). An improved prediction of catalytic residues in enzyme structures. Protein Eng. Des. Sel..

[57] Jones D.T. (2007). Improving the accuracy of transmembrane protein topology prediction using evolutionary information. Bioinformatics.

[58] Karypis G. (2006). YASSPP: Better kernels and coding schemes lead to improvements in protein secondary structure prediction. Proteins.

[59] Kabsch W., Sander C. (1983). Dictionary of protein secondary structure: Pattern recognition of hydrogen-bonded and geometrical features. Biopolymers.

[60] Carter P., Andersen C.A., Rost B. (2003). DSSPcont: Continuous secondary structure assignments for proteins. Nucleic Acids Res..

[61] Tjong H., Zhou H.X. (2007). DISPLAR: An accurate method for predicting DNA-binding sites on protein surfaces. Nucleic Acids Res..

[62] SJ H., JM T. (1993). NACCESS Computer program. Department of Biochemistry and Molecular Biology.

[63] Faucher J., Pliska V. (1983). Hydrophobic parameters pi of amino acid side chains from the partitioning of *N*-acetyl-amino-acid amides. Eur. J. Med. Chem..

[64] Kyte J., Doolittle R.F. (1982). A simple method for displaying the hydropathic character of a protein. J. Mol. Biol..

[65] Tsuchiya Y., Kinoshita K., Nakamura H. (2004). Structure-based prediction of DNA-binding sites on proteins using the empirical preference of electrostatic potential and the shape of molecular surfaces. Proteins.

[66] Shazman S., Celniker G., Haber O., Glaser F., Mandel-Gutfreund Y. (2007). Patch Finder Plus (PFplus): A web server for extracting and displaying positive electrostatic patches on protein surfaces. Nucleic Acids Res..

[67] Brooks B.R., Bruccoleri R.E., Olafson B.D., States D.J., Swaminathan S., Karplus M. (1983). *CHARMM—*A program for macromolecular energy, minimization and dynamics calculations. J. Comput. Chem..

[68] Ahmad S., Sarai A. (2004). Moment-based prediction of DNA-binding proteins. J. Mol. Biol..

[69] Sali A., Blundell T.L. (1993). Comparative protein modelling by satisfaction of spatial restraints. J. Mol. Biol..

[70] Fischer J.D., Mayer C.E., Soding J. (2008). Prediction of protein functional residues from sequence by probability density estimation. Bioinformatics.

[71] Ding X.M., Pan X.Y., Xu C., Shen H.B. (2010). Computational prediction of DNA–protein interactions: A review. Curr. Comput. Aided Drug Des..

[72] Bhardwaj N., Langlois R.E., Zhao G., Lu H. (2005). Kernel-based machine learning protocol for predicting DNA-binding proteins. Nucleic Acids Res..

[73] Cortes C., Vapnik V. (1995). Support-vector networks. Mach. Learn..

[74] Chu W.Y., Huang Y.F., Huang C.C., Cheng Y.S., Huang C.K., Oyang Y.J. (2009). ProteDNA: A sequence-based predictor of sequence-specific DNA-binding residues in transcription factors. Nucleic Acids Res..

[75] Bhardwaj N., Langlois R., Zhao G., Lu H. (2005). Structure based prediction of binding residues on DNA-binding proteins. Conf. Proc. IEEE Eng. Med. Biol. Soc..

[76] Shao X., Tian Y., Wu L., Wang Y., Jing L., Deng N. (2009). Predicting DNA- and RNA-binding proteins from sequences with kernel methods. J. Theor. Biol..

[77] Sun Y.F., Fan X.D., Li Y.D. (2003). Identifying splicing sites in eukaryotic RNA: Support vector machine approach. Comput. Biol. Med..

[78] Lu Y., Wang X., Chen X., Zhao G. (2013). Computational methods for DNA-binding protein and binding residue prediction. Protein Pept. Lett..

[79] Lou W., Wang X., Chen F., Chen Y., Jiang B., Zhang H. (2014). Sequence based prediction of DNA-binding proteins based on hybrid feature selection using random forest and Gaussian naive Bayes. PLoS One.

[80] Wang L., Yang M.Q., Yang J.Y. (2009). Prediction of DNA-binding residues from protein sequence information using random forests. BMC Genomics.

[81] Ho T.K. (2002). A Data complexity analysis of comparative advantages of decision forest constructors. Pattern Anal. Appl..

[82] Martin-Galiano A.J., Smialowski P., Frishman D. (2008). Predicting experimental properties of integral membrane proteins by a naive Bayes approach. Proteins.

[83] Rhodes D.R., Tomlins S.A., Varambally S., Mahavisno V., Barrette T., Kalyana-Sundaram S., Ghosh D., Pandey A., Chinnaiyan A.M. (2005). Probabilistic model of the human protein–protein interaction network. Nat. Biotechnol..

[84] Ozbek P., Soner S., Erman B., Haliloglu T. (2010). DNABINDPROT: Fluctuation-based predictor of DNA-binding residues within a network of interacting residues. Nucleic Acids Res..

[85] Bujnicki J.M., Elofsson A., Fischer D., Rychlewski L. (2001). LiveBench-1: Continuous benchmarking of protein structure prediction servers. Protein Sci..

[86] Huang B., Schroeder M. (2008). Using protein binding site prediction to improve protein docking. Gene.

[87] Ferrer-Costa C., Shanahan H.P., Jones S., Thornton J.M. (2005). HTHquery: A method for detecting DNA-binding proteins with a helix-turn-helix structural motif. Bioinformatics.

[88] Wang L., Brown S.J. (2006). BindN: A web-based tool for efficient prediction of DNA and RNA binding sites in amino acid sequences. Nucleic Acids Res..

[89] Nimrod G., Schushan M., Szilagyi A., Leslie C., Ben-Tal N. (2010). iDBPs: A web server for the identification of DNA binding proteins. Bioinformatics.

[90] Zhang Y., Xu J., Zheng W., Zhang C., Qiu X., Chen K., Ruan J. (2014). newDNA-Prot: Prediction of DNA-binding proteins by employing support vector machine and a comprehensive sequence representation. Comput. Biol. Chem..

[91] Liu R., Hu J. (2013). DNABind: A hybrid algorithm for structure-based prediction of DNA-binding residues by combining machine learning- and template-based approaches. Proteins.

[92] Ma X., Guo J., Liu H.D., Xie J.M., Sun X. (2012). Sequence-based prediction of DNA-binding residues in proteins with conservation and correlation information. IEEE/ACM Trans. Comput. Biol. Bioinform..

[93] Chen Y.C., Wright J.D., Lim C. (2012). DR_bind: A web server for predicting DNA-binding residues from the protein structure based on electrostatics, evolution and geometry. Nucleic Acids Res..

[94] Matthew S., Tianyin Z., Lin Y., Ana C.D.M., Raluca G., Remo R. (2014). Absence of a simple code: How transcription factors read the genome. Trends Biochem. Sci..

[95] Yang L., Zhou T.Y., Iris D., Anthony M., Wyeth W.W., Raluca G., Remo R. (2014). NAR breakthrough article: TFBSshape: A motif database for DNA shape features of transcription factor binding sites. Nucleic Acids Res..

[96] Ghersi D., Sanchez R. (2009). Improving accuracy and efficiency of blind protein-ligand docking by focusing on predicted binding sites. Proteins.

[97] Kauffman C., Rangwala H., Karypis G. (2008). Improving homology models for protein-ligand binding sites. Comput. Syst. Bioinform. Conf..

[98] Schroder A., Eichner J., Supper J., Eichner J., Wanke D., Henneges C., Zell A. (2010). Predicting DNA-binding specificities of eukaryotic transcription factors. PLoS One.

[99] Cai Y., He J., Li X., Lu L., Yang X., Feng K., Lu W., Kong X. (2009). A novel computational approach to predict transcription factor DNA binding preference. J. Proteome Res..

[100] Qian Z., Lu L., Liu X., Cai Y.D., Li Y. (2007). An approach to predict transcription factor DNA binding site specificity based upon gene and transcription factor functional categorization. Bioinformatics.

[101] Qian Z., Cai Y.D., Li Y. (2006). A novel computational method to predict transcription factor DNA binding preference. Biochem. Biophys. Res. Commun..

[102] Zhu L., Yang J., Song J.N., Chou K.C., Shen H.B. (2010). Improving the accuracy of predicting disulfide connectivity by feature selection. J. Comput. Chem..

